# Tubular and Spherical SiO_2_ Obtained by Sol Gel Method for Lipase Immobilization and Enzymatic Activity

**DOI:** 10.3390/molecules23061362

**Published:** 2018-06-05

**Authors:** Crina Anastasescu, Silviu Preda, Adriana Rusu, Dana Culita, Gabriel Plavan, Stefan Strungaru, Jose Maria Calderon-Moreno, Cornel Munteanu, Catalina Gifu, Mirela Enache, Radu Socoteanu, Daniel Angelescu, Mihai Anastasescu, Mariuca Gartner, Ioan Balint, Maria Zaharescu

**Affiliations:** 1 “Ilie Murgulescu” Institute of Physical Chemistry of the Romanian Academy, 060021 Bucharest, Romania; canastasescu@icf.ro (C.A.); predas@icf.ro (S.P.); arusu@icf.ro (A.R.); dculita@icf.ro (D.C.); josecalderonmoreno@yahoo.com (J.M.C.-M.); munteanuc@icf.ro (C.M.); menache@icf.ro (M.E.); dangelescu@hotmail.com (D.A.); mgartner@icf.ro (M.G.); ibalint@icf.ro (I.B.); 2 Faculty of Biology, “Alexandru Ioan Cuza” University, 700505 Iasi, Romania; gabriel.plavan@uaic.ro (G.P.); strungaru_stefan@yahoo.com (S.S.); 3 National Research and Development Institute for Chemistry and Petrochemistry-ICECHIM, 060021 Bucharest, Romania; gifu_ioanacatalina@yahoo.com

**Keywords:** SiO_2_, tubular and spherical morphology, lipase immobilization, enzymatic catalysis

## Abstract

A wide range of hybrid biomaterials has been designed in order to sustain bioremediation processes by associating sol-gel SiO_2_ matrices with various biologically active compounds (enzymes, antibodies). SiO_2_ is a widespread, chemically stable and non-toxic material; thus, the immobilization of enzymes on silica may lead to improving the efficiency of biocatalysts in terms of endurance and economic costs. Our present work explores the potential of different hybrid morphologies, based on hollow tubes and solid spheres of amorphous SiO_2_, for enzyme immobilization and the development of competitive biocatalysts. The synthesis protocol and structural characterization of spherical and tubular SiO_2_ obtained by the sol gel method were fully investigated in connection with the subsequent immobilization of lipase from *Rhizopus orizae*. The immobilization is conducted at pH 6, lower than the isoelectric point of lipase and higher than the isoelectric point of silica, which is meant to sustain the physical interactions of the enzyme with the SiO_2_ matrix. The morphological, textural and surface properties of spherical and tubular SiO_2_ were investigated by SEM, nitrogen sorption, and electrokinetic potential measurements, while the formation and characterization of hybrid organic-inorganic complexes were studied by UV-VIS, FTIR-ATR and fluorescence spectroscopy. The highest degree of enzyme immobilization (as depicted from total organic carbon) was achieved for tubular morphology and the hydrolysis of p-nitrophenyl acetate was used as an enzymatic model reaction conducted in the presence of hybrid lipase–SiO_2_ complex.

## 1. Introduction

Recently, intensive research has been dedicated to the development of new and competitive technologies based on engineered materials which are utilized in competitive industrial processes, advanced healthcare and environmental protective systems. Accordingly, a wide range of multifunctional materials, including SiO_2_ matrices, have been designed for catalytic/photocatalytic oxidative degradation of organic compounds assimilated to environmental pollutants, and also for bioremediation processes like enzymatic treatments of air, water, and soil [1,2].

Silica is an extensively studied, widely used material, especially as a support for various active phases and also as part of hybrid organic-inorganic complexes, together with enzymes, antibodies, biological markers [3].

In general, the immobilization of enzymes onto inorganic supports is undertaken to preserve and select the ultimate catalytic performance for a target reaction, while also searching for an increase in enzyme life time, activity and endurance regarding the surrounding parameters, pH and temperature [4].Additionally, better recovery and re-usability of the enzyme can be achieved by immobilization, which is particularly desirable for enzymatic processes involved in technologies with high economic impact. Lipase is widely used for catalytic reactions such as esterification, transesterification, and biodiesel processing [4,5,6].

In our previous papers we demonstrated that the photo/catalytic mineralization of some organic compounds (oxalic and formic acid) can be successfully achieved on platinum modified tubular SiO_2_ which work as microreactors [7,8].

Approaches that conduct to hybrid organic-inorganic structures usually focus on the physical interactions between enzymes and inorganic surfaces, encapsulation of enzymes in the inorganic matrix during or post synthesis, or its covalent attachment to the support [9]. The present study exploits the already known advantages of tubular geometry, that is, high surface area, tunable porosity, ability to bind distinctively chemical or biological active compounds to internal or external surfaces, by expanding them to the still unexplored but captivating area of biocatalysts. This paper emphasizes the significant potential of amorphous SiO_2_ with spherical and tubular morphologies to immobilize lipase originating from *Rhizopus orizae* by examining the physical interactions that reveal the different physico-chemical and functional characteristics of the spherical and tubular SiO_2_ together with their derivative hybrids.

The great majority of the studies which have investigated the development of derivatives starting from the immobilization of enzymes, have focused on porous silica (mono/bimodal porosity) with its high surface area [10], and have aimed at tailoring the hydrophilic/hydrophobic characteristics of silica surfaces (the last being especially required for lipase immobilization) [11,12] while investigating the deposition of appropriate chemical species that are able to strongly bind the enzymes [13,14]. Although the derivatives based on mesoporous silica materials have been intensively investigated up to now, there is still room for designing new and versatile SiO_2_ matrices as a host of valuable enzymes. Our work examines the different behaviors of silica with different morphologies (two types of nanotubes and spherical particles) towards lipase adsorption and its catalytic activity after immobilization.

The aims of this study are as follows:

(i) To determine the optimal parameters of SiO_2_ synthesis by the sol gel method assisted by an in situ generated template, in correlation with the required conditions to maximize the degree of lipase adsorption onto the silica spheres and tubes surface; to assess whether the internal or external surface of nanotubes are appropriate to link lipase and to correlate the internal diameter with the efficiency of lipase immobilization.

Accordingly, scanning electron microscopy (SEM), N_2_ sorption and infra-red spectroscopy (FT-IR) analyses were carried out.

(ii) To demonstrate the development of the lipase–SiO_2_ hybrid structure. For this purpose, characterization was carried out by FT-IR ATR, spectrofluorescence, electrokinetic potential and total organic carbon (TOC) measurements.

(iii) To test the activity of hybrid material (lipase/SiO_2_) for the hydrolysis reaction of p-nitrophenyl acetate (p-NPA) carried out in the presence of lipase immobilized onto SiO_2_ samples.

## 2. Results and Discussion

### 2.1. Scanning Electron Microscopy (SEM)

**Figure 1:**
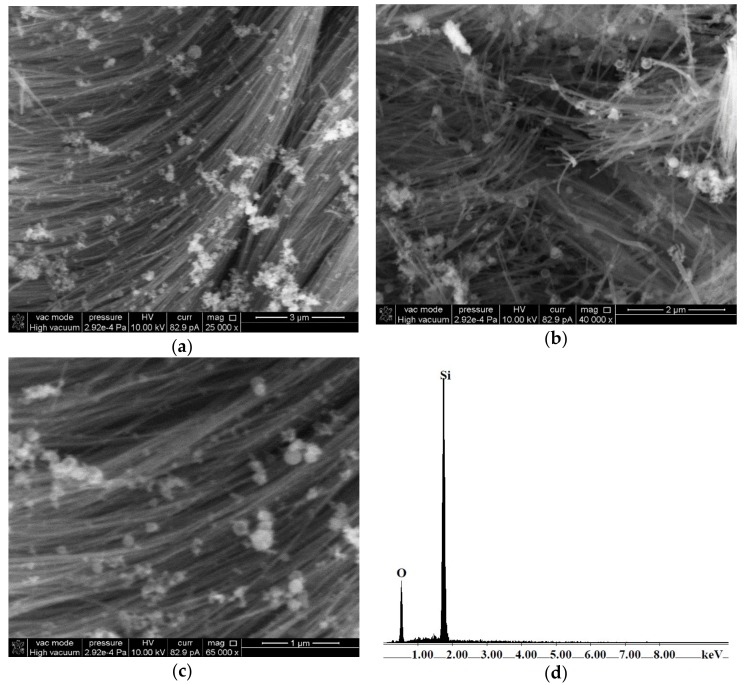
(**a**–**c**) SEM micrographs at different magnifications and (**d**) EDX spectrum of SiO_2_-t.

**Figure 2:**
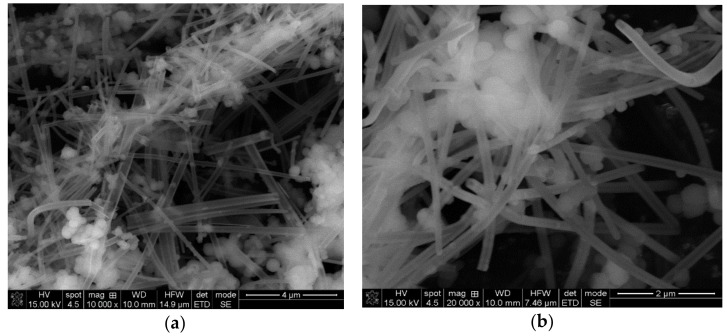
(**a**,**b**) SEM micrographs of SiO_2_-T at different magnifications, showing the hollow tubes structure.

SiO_2_ solid carriers with tubular morphologies: (i) small diameter (<100 nm) nanotubes, named as SiO_2_-t (Figure 1), and (ii) larger diameter (>200 nm) tubes, named as SiO_2_-T (Figure 2) were prepared by a sol-gel method. Both materials contain a small fraction of non-tubular hollow quasi-spherical SiO_2_ particles, mixed with the tubes, accounting for over 95% of the material. The co-existence of rounded particles as a minority phase is a characteristic of tubular SiO_2_ materials prepared by sol-gel method, as previously reported in the literature [15,16].

SEM micrographs of SiO_2_-t at different magnification scales (Figure 1) highlight the presence of thin, very long (tens of microns) nanotubes with open ends. Thin SiO_2_-t nanotubes have external diameters typically ranging between 20 and 50 nm, aggregating in flexible bunches (Figure 1). The BET measurements showed that this morphology can be assimilated to the hollow fibers. Along with bunches of thin nanotubes with homogeneous diameter and length, a few shorter, straight tubes with larger diameters (up to 100 nm) can also be observed, along with the above mentioned hollow spherical particles (with an external diameter of 50–200 nm). EDX analysis confirms the presence of Si and O as the only constituent elements.

SEM micrographs of SiO_2_-T (Figure 2) reveal the presence of larger tubes, with diameters between 100 and 400 nm and a typical diameter of ~200 nm.

The tubes internal walls of about 50 nm thickness appear clearly resolved (Figure 2a). Spherical SiO_2_ particles are also present in higher proportion (~30%) and with a larger radius compared with those observed in SiO_2_-t. Tubes in SiO_2_-T have lengths of a few microns, are mostly straight and randomly oriented. The open ends shape and the clearly resolved internal walls indicate a tendency towards a rectangular shape of the tubes.

**Figure 3:**
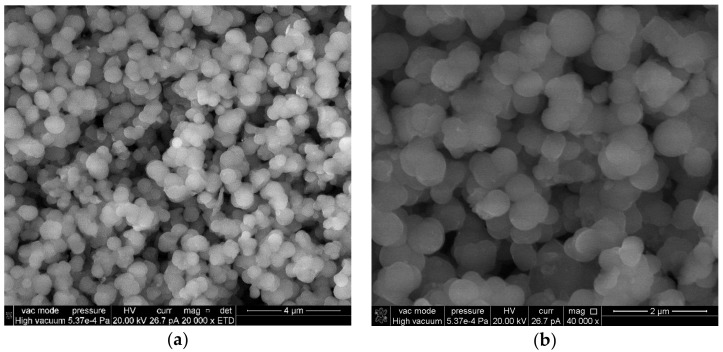
(**a,b**) SEM micrographs of SiO_2_-S at different magnifications showing the spherical morphology.

In order to investigate the influence of the morphology on the catalytic activity of derived hybrid structures, a third SiO_2_ material containing only spherical particles (denoted as SiO_2_-S) was prepared by the sol-gel method. Spherical particles in SiO_2_-S (Figure 3) are solid and have diameters of a few hundreds nm (200–600 nm).

### 2.2. Nitrogen Sorption Measurements

**Figure 4:**
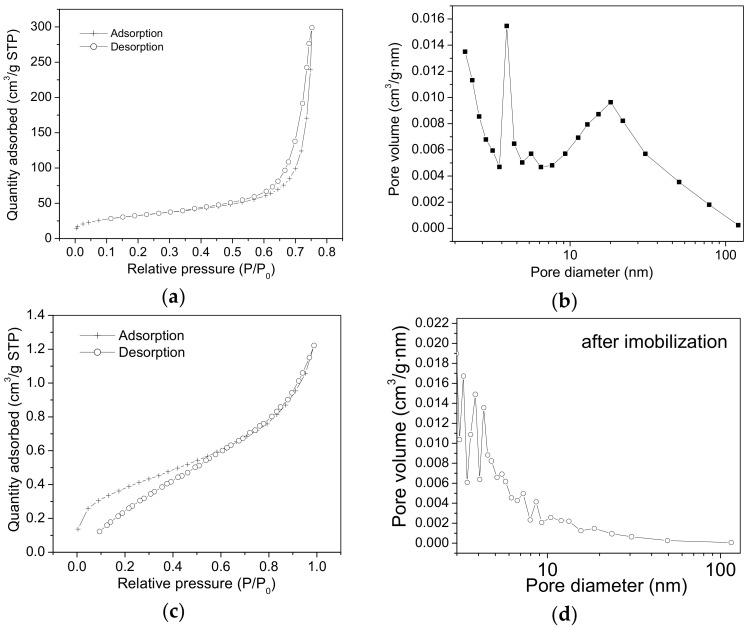
N_2_ adsorption-desorption isotherms (**a**) and the pore size distribution obtained from the desorption branch (**b**) for the SiO_2_-t samples before lipase immobilization; (**c**,**d**)—the same curves recorded after lipase immobilization.

According to BET measurements, the surface area (S_BET_) value of the SiO_2_-t samples is 107.83 m^2^/g (Figure 4a). This is an intermediate value between our previously obtained values for SiO_2_ powder containing nanotubes with diameters of about 200–400 nm, characterized by a value of S_BET_ ~ 300 m^2^/g [8] and much higher than that of S_BET_ ~ 5 m^2^/g corresponding to a powder composed of larger tubes with diameters up to 2 μm [17].

The adsorption and desorption branches are almost parallel, which indicates the presence of cylindrical pores, with open ends. The pore size distribution is broad (Figure 4b), ranging between 10 and 100 nm. In correlation with SEM images (Figure 1), it can be seen that for most of the tubes the internal diameter measures 10–50 nm. Additionally, a high maximum point located at ~3 nm (Figure 4b) could be generated by the mesoporosity of the nanotubes walls. 

These textural characteristics seem to be appropriate for lipase adsorption onto both the external and internal surfaces of the SiO_2_-t nanotubes.

With regard to the lipase adsorption onto the internal surface of the SiO_2_-t nanotubes (diameter of lipase ~50 Å), the presence of the enzymes inside the nanotubes core could be assimilated to a state of “confinement” in the SiO_2_ tubular matrix which should provide increased stability in the newly formed hybrid structure, [18] including a diminished degree of lipase leaching in solution post immobilization. At the same time, the known limitations resulting from the diffusion in narrow pores/voids, should be diminished here because the tubes have open ends, regular geometry and large diameters.

Figure 4c,d show the strongly modified textural properties of SiO_2_-t after lipase immobilization. The main broad peak ranging between 10 and 100 nm is eliminated, which indicates that the lipase adsorption onto these thin tubes is mainly accomplished by the lipase immobilization inside their core.

**Figure 5:**
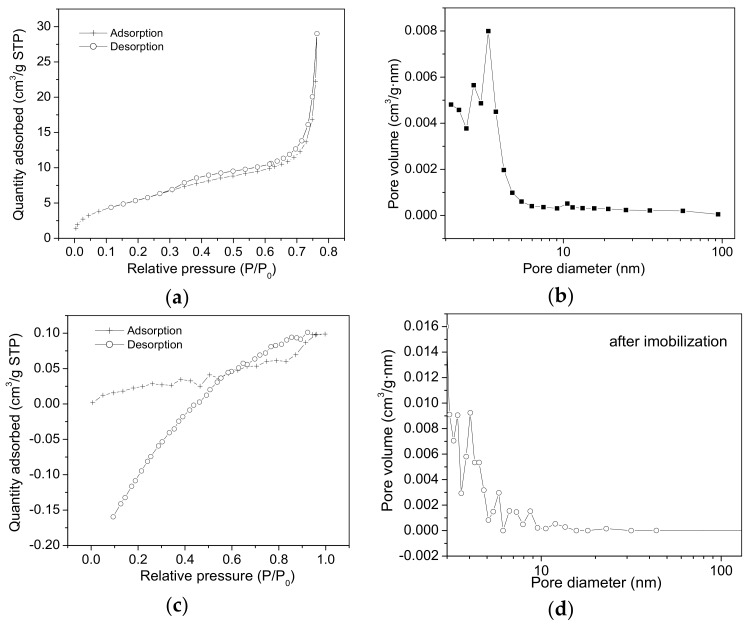
N_2_ adsorption-desorption isotherms (**a**) and the pore size distribution obtained from the desorption branch (**b**) for the SiO_2_-T samples before lipase immobilization; (**c**,**d**)—the same curves recorded after lipase immobilization.

Figure 5b reveals the textural characteristics of the SiO_2_-T sample, with its significantly smaller surface area (S_BET_) of about 18 m^2^/g and quite uniform mesoporosity of 3 nm. Clearly, this represents the diameter of pores from the wall structure, the internal diameters of nanotubes (~200 nm) could not be depicted from these measurements, but certainly the empty core of the nanotubes (Figure 2) would be fully accessible for lipase adsorption. It is also presumed that the adsorption of lipase from *Rhizopus orizae* (with a diameter of ~50 Å) hardly takes place in the small mesopores of walls and thus, these reduced spaces probably induce some conformational and flexibility restrictions which could mean further denaturation of the enzymes.

Figure 5c,d illustrate the adsorption isotherm and the desorption branch for the SiO_2_-T sample after lipase immobilization. Unlike the SiO_2_-t sample, the pore size distribution depicted in the desorption branch does not seem to be drastically modified. However, the isotherm shows an obvious change, which could be related to massive adsorption inside the tubes, which have diameters bigger than 100 nm, placing them in the macropore domain.

**Figure 6:**
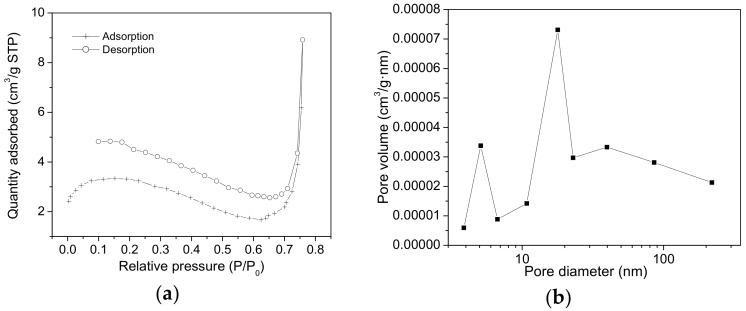
N_2_ adsorption-desorption isotherms (**a**) and the pore size distribution obtained from the adsorption branch (**b**) for the SiO_2_-S samples before enzymatic immobilization.

Figure 6 shows that the spherical sample (SiO_2_-S) has a small surface area (S_BET_ ~ 14 m^2^/g) and irregular porosity, spanning a large domain, between 3–5 nm and 10–30 nm. The second value obtained due to the sphere packing may allow the immobilization of the enzymes but this structure could induce massive post immobilization leaching.

Taking into account the information derived from Figure 4 and Figure 5, we predicted a higher degree of lipase immobilization for the tubular samples than for the spherical ones. Additionally, a survey of the literature revealed similar efforts to develop feasible biocatalysts, by triggering the immobilization of an enzyme with high volumetric activity (U/g) on a solid matrix with high payload. This was defined by Cao [4] as an appropriate ratio (0.1–0.2) of immobilized enzyme and the mass of used support.

In our opinion, the optimized SiO_2_ tubular materials obtained by the sol gel method, especially those with high surface area and diameters up to 100 nm, should provide a potentially solid carrier for a valuable immobilized biocatalyst.

### 2.3. Fourier Transformed Infra-Red (FTIR) Spectroscopy

**Figure 7:**
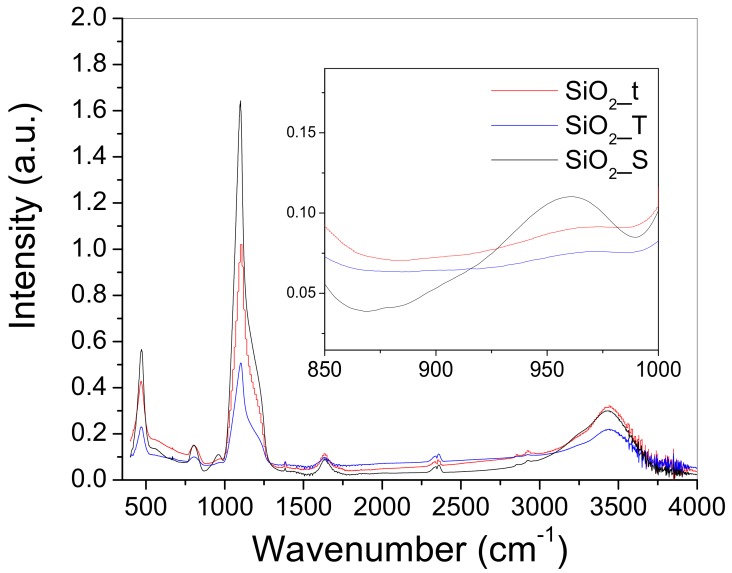
Comparative FTIR spectra of SiO_2_ samples, (prior to lipase immobilization) with different morphology: thin tubes (SiO_2_-t), bigger tubes (SiO_2_-T) and spherical particles (SiO_2_-S). Inset shows in detail the shoulder appearing at ~960 cm^−1^.

FTIR spectra (Figure 7) show the comparative features of the main vibration bands, and that the chemical “fingerprint” is in fact identical for the SiO_2_ samples with different morphologies. Although, small differences appear in the intensity of the bands and for the morphology-dependent surface density of OH groups, which may be further related to the different adsorption and activation degree of lipase on the surface of SiO_2_.

The common bands are further ascribed according to the literature and our previous work as follows.

The sharp and well defined peak from 1124 cm^−1^ can be assigned to Si-O stretching in SiO_2_ [7]. It can also be observed that this peak has a shoulder close to 1200 cm^−1^ which is attributed to the asymmetric vibration of Si-O-Si [19].

The peaks situated at 847 cm^−1^ and 484 cm^−1^ are determined by the ν_s(Si-O-Si)_ and *δ*_(Si-O-Si)_, respectively [20].

The shoulder located at ~980–960 cm^−1^ assigned to the presence of silanol groups (Si-OH) [21] is well defined only for the sample with spherical morphology (SiO_2_-S), in the case of tubular samples (SiO_2_-T and SiO_2_-t) this is missing. Furthermore, the presence of structural and free OH groups, along with adsorbed water, indicated by the broad bands appearing at 3350–3600 cm^−1^ [16,22,23,24] is slightly decreased for the SiO_2_-T sample and enhanced for SiO_2_-t.

In general, the intrinsic density of OH groups is a material dependent particularity derived from its synthesis protocol. Also, the strong influence of the intrinsic density of OH groups on the photo/catalytic activity of various materials [25] has been fully demonstrated. Likewise, Sun et al. [26] demonstrated the connection between the sterilizing and antimicrobial activity of SiO_2_-TiO_2_ films to the action of OH groups. In our case, their presence favors the formation of hydrogen bounds between the inorganic substrate and some appropriate functional groups of enzymes, increasing in this way the degree of enzyme immobilization [27].

Our data based on density of OH groups and adsorbed water indicates the following sequence in decrease of hydrophobicity: SiO_2_-T > SiO_2_-t > SiO_2_-S. Subsequently, the activity of immobilized lipase could be discussed in what comes from this point of view, too.

All of the previously discussed data are summarized in Table 1.

### 2.4. FTIR-ATR Spectroscopy Performed on Hybrid Materials

**Figure 8:**
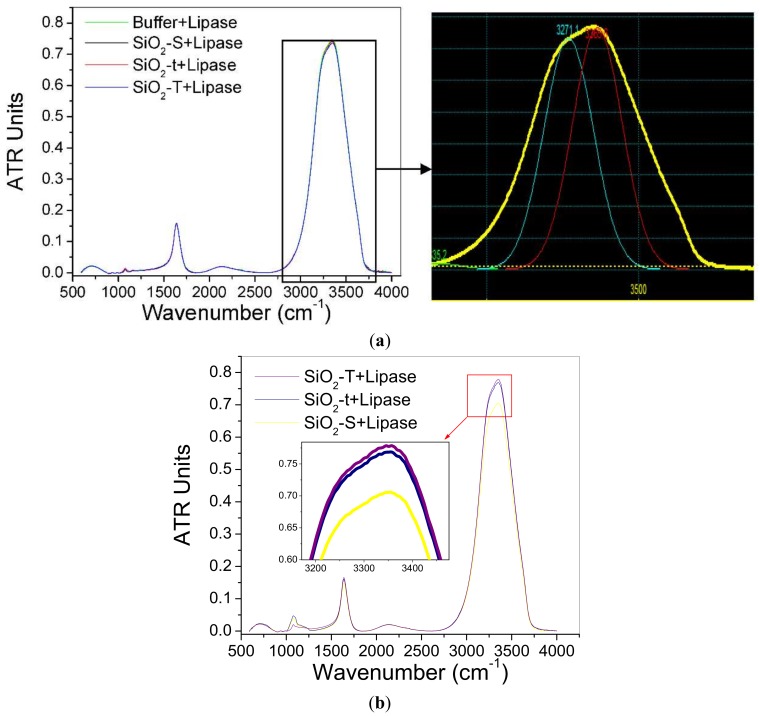
FTIR-ATR spectra for lipase and SiO_2_ samples in K_2_HPO_4_ buffer solution after immobilization: (**a**) recorded from supernatant—with two Gaussian signals deconvolution (Peakfit program) revealing the 3271 cm^−1^ peak, and (**b**) from solid powder.

The acquisition mode was Double Sided, Forward-Backward with no correlation mode. For FT (Fourier Transform), the Blackman-Harris apodization function was used; most other settings are automatically standardized. The spectra were normalized and atmospherically (H_2_O vapors, CO_2_) compensated. Figure 8a,b show the spectra obtained under the above-mentioned conditions. Multiple spectra were normalized to each other.

The specific vibration bands of the target component bounds, lipase, are fully identified both in the supernatant (liquid) phase (Figure 8a) and even better in the solid SiO_2_ matrices after the lipase immobilization (Figure 8b).

The spectral IR general profile is characteristic for lipase according to the literature [29]; the characteristic bands are presented in Table 2. The most important signal, as weight, in the lipase spectrum is that provided by the amide group I, due to the vibrations of the NH group, first of the deformation (*δ*) of 1635 cm^−1^ and the stretching (γ) from 3271 cm^−1^. Figure 8a shows the decreasing intensity of the lipase signals (3271 cm^−1^) from the supernatant solutions, composed of the following systems: Potassium phosphate buffer–Lipase starting solution > Potassium phosphate buffer–Lipase (SiO_2_-S) > Potassium phosphate buffer–Lipase (SiO_2_-t); Potassium phosphate buffer–Lipase (SiO_2_-T).

The spectra shown in Figure 8b are in line with the previous ones, the greatest lipase signals (3271 cm^−1^) correlate with the lipase-SiO_2_-T complex (which had the smaller concentration of lipase in supernatant), according to the sequence: SiO_2_-T > SiO_2_-t > SiO_2_-S.

The lack or attenuation up to the baseline of the other signals, especially amide II and III, may be due to low concentrations of lipase. The common signal at 1635 cm^−1^ may indicate a unitary system containing the lipase, as shown in Figure 8.

Clearly, the immobilization of lipase on the SiO_2_ surfaces can be evaluated from the high intensity peaks recorded, especially from solid samples, even at lower lipase concentrations.

### 2.5. Photoluminescence (PL)

**Figure 9:**
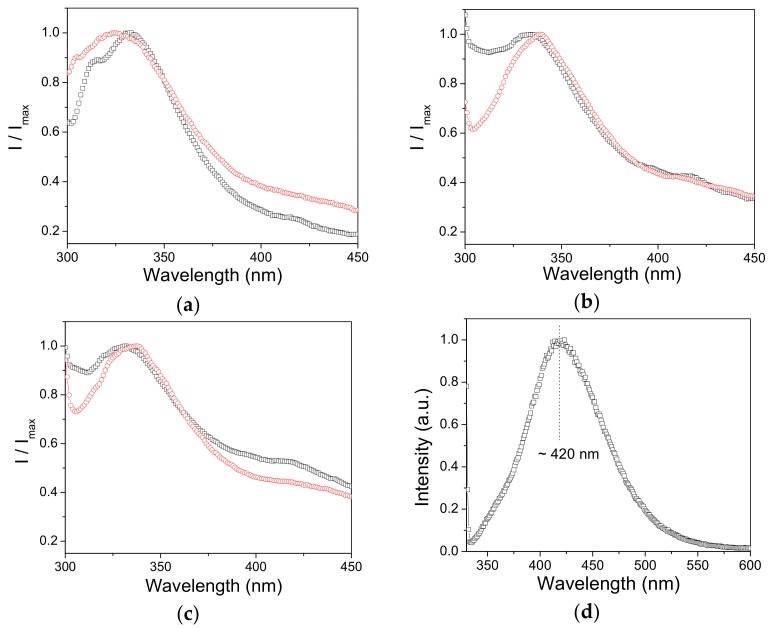
Normalized fluorescence of (о) free and (□) nanoparticle-bound lipase of the samples (**a**) SiO_2_-T, (**b**) SiO_2_-S and (**c**) SiO_2_-t, for λ_exc_ = 270 nm. (**d**) Fluorescence spectrum registered for SiO_2_-t bound lipase for λ_exc_ = 320 nm.

Intrinsic fluorescence of lipase has been assessed to examine the enzyme-nanoparticle complexation. The normalized emission spectra of lipase in the presence of SiO_2_ nanoparticles of three different morphologies are shown in Figure 9a–c.

The most noteworthy feature is the broad band exhibiting a maximum at ~332 nm, thus indicating the similar protein conformation adopted at the SiO_2_ surface despite the varied morphology.

To gain further insights into the complexation mechanism, Figure 9a–c illustrates the emission spectra of the free lipase. In the case of samples SiO_2_-S and SiO_2_-t, it appears that the lipase emission undergoes a hypsochromic shift of about 5 nm upon binding. This displacement in maximum emission suggests conformational changes such that Trp residue experiences a more hydrophobic environment when lipase becomes immobilized. As for the SiO_2_-T sample, a red shift of about 5 nm is noted, which likely originates from the different environmental conditions experienced in the precursor system. It should be also mentioned that the fluorescence intensity does not decrease pronouncedly upon enzyme binding, and the lack of a significant fluorescence quenching is in contrast with previous investigations of lipase binding to functionalized gold nanoparticles [30]. When sample excitation is carried out at λ_exc_ = 320 nm, the intrinsic fluorescence of the SiO_2_-t matrix can be envisaged, as shown in Figure 9d where the enzyme fluorescence is noted as a shallow shoulder at ~360 nm. The broad photoluminescence centered at 420 nm is in line with the emission observed recently at ~400 nm for silica tubular walls by Anastasescu et al. [17]. Note that the latter morphology resulted in an additional band at ~500 nm, and the absence of this band for the present SiO_2_ systems could be due to the differences in the surface defects. Thus, it can be concluded that the photoluminescence investigation suggested a lipase immobilization into a new conformational state onto the emitting SiO_2_-t matrix.

### 2.6. Total Organic Carbon (TOC)

**Figure 10:**
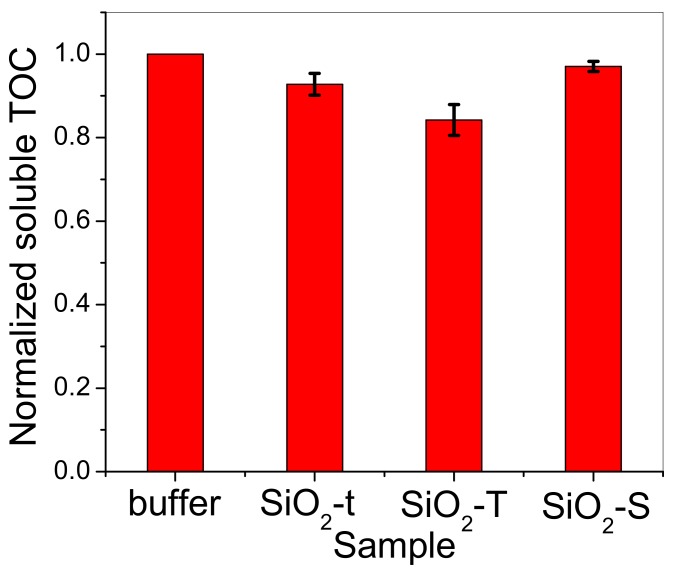
Soluble TOC values normalized to initial lipase concentration in buffer solution, registered for triplicate samples (three specimens for each morphology).

Figure 10 presents the normalized soluble TOC measurements performed in triplicate (see the error bar which represents the resulting confidence interval for each morphology) from supernatant solutions, relative to the initial concentration of dissolved lipase in buffer solution (0.8 mg/mL), after immobilization of the lipase on SiO_2_ matrices with different morphologies.

**Figure 11:**
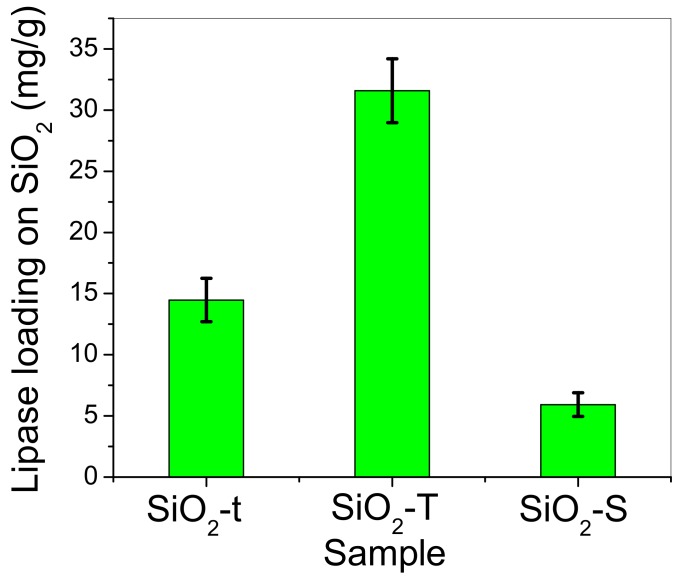
The different quantitative lipase loading (including the error bars of each sample) on SiO_2_ matrices, obtained for a triplicate set of samples.

Figure 10 indicates that the highest decrease in lipase concentration from the supernatant occurs for tubular morphologies (SiO_2_-t and SiO_2_-T), especially for the sample containing the bigger tubes (SiO_2_-T). Previous results from Figure 10 were used to evaluate the lipase loading on the solid SiO_2_ matrices. Figure 11 illustrates the estimated lipase loading capacity of each SiO_2_ sample, including the error bar calculated for each sample. Interestingly, the highest loading capacity is observed for the large tubes (SiO_2_-T) and the lowest for the spherical morphology (SiO_2_-S).

### 2.7. The Electro-Kinetic Potential

The electro-kinetic potential (ξ) was determined in order to decipher and evaluate the electrostatic interactions between the silica surface and enzyme, this being a key parameter for a high degree of lipase adsorption onto the silica surface. The literature suggests that the isoelectric point of silica is at pH around 3 and that lipase from *Rhizopus orizae* has a pH 7.6 [31]. Our enzymatic immobilization tests were conducted at pH 6.3, where SiO_2_ is negatively charged and lipase bears a slightly positive charge. These experimental conditions should ensure electrostatic attraction between SiO_2_ and lipase. These measurements were done for: (i) the pristine SiO_2_ powders dispersed in water, (ii) immobilized lipase onto SiO_2_ powders re-dispersed in water and (iii) SiO_2_ powders dispersed in a solution of lipase and potassium phosphate (buffer), at 25 °C.

**Figure 12:**
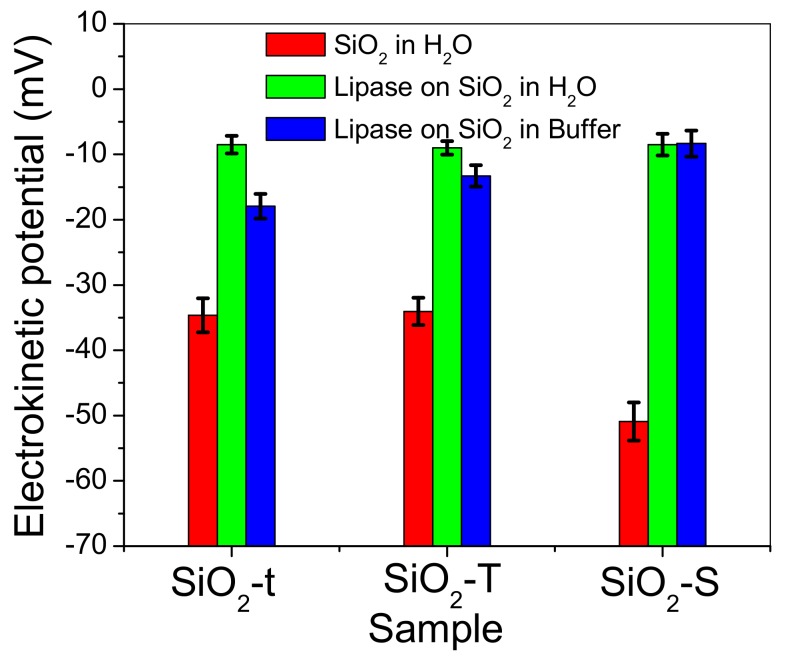
The electrokinetic potential of the SiO_2_ matrices and their derivative hybrid systems, registered for a triplicate set of samples.

Figure 12 clearly illustrates the negative charge of all three SiO_2_ samples in aqueous medium, the highest negative value of the electrokinetic potential being registered for spherical silica (SiO_2_-S). The experiments were performed in triplicate, and the error bars represent the deviation for each sample (three specimens for each morphology).

The tubular silica samples (SiO_2_-T and SiO_2_-t) dispersed in water show the same value of ~−34 mV for electrokinetic potential, which should be similar to the degree of lipase adsorption, if we assimilate it with the variations in electrokinetic potential. Accordingly, it can be seen that the immobilization of lipase onto SiO_2_ surfaces leads to a significant decrease in ξ potential, which amounts to about −8 mV irrespective of the SiO_2_ morphology. In addition, ξ potential after lipase immobilization seems to be poorly influenced by the ionic strength, in particular for SiO_2_-T and SiO_2_-S nanoparticles, a feature denoting highly coupled electrostatic systems.

### 2.8. Catalytic Activity

The hydrolysis of p-nitrophenyl acetate (p-NPA) is often used as a model reaction to investigate enzymatic kinetics [32].

The hydrolysis reaction of p-nitrophenyl acetate strongly depends on the solvent choice [33] but especially on the pH of the medium. According to previous data, the identified product of the hydrolysis reaction is p-nitrophenol (at pK_a_ 7.2) and this results in UV-VIS measurements in the range of 400–410 nm (ε_molar_ 14,200 M^−1^ cm^−1^) [27,32,34,35].

**Figure 13:**
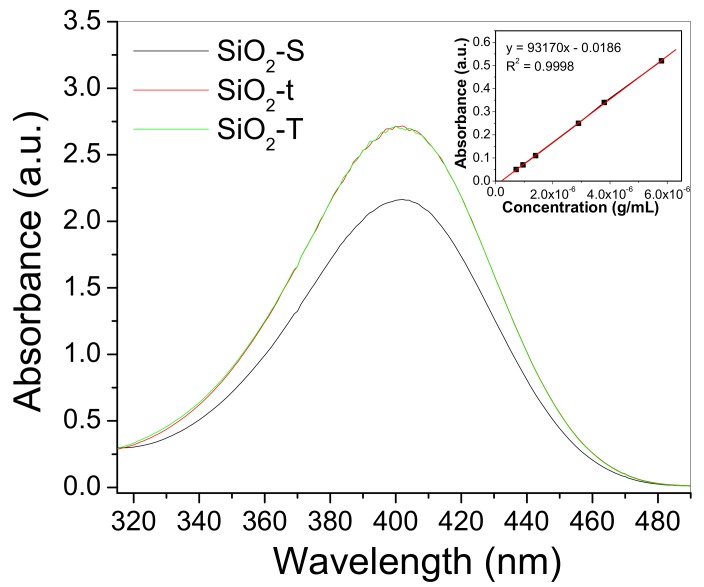
p-nitrophenol acetate (p-NPA) hydrolysis product after 1 h of incubation; Inset: calibration of p-NP concentration from UV-VIS.

**Figure 14:**
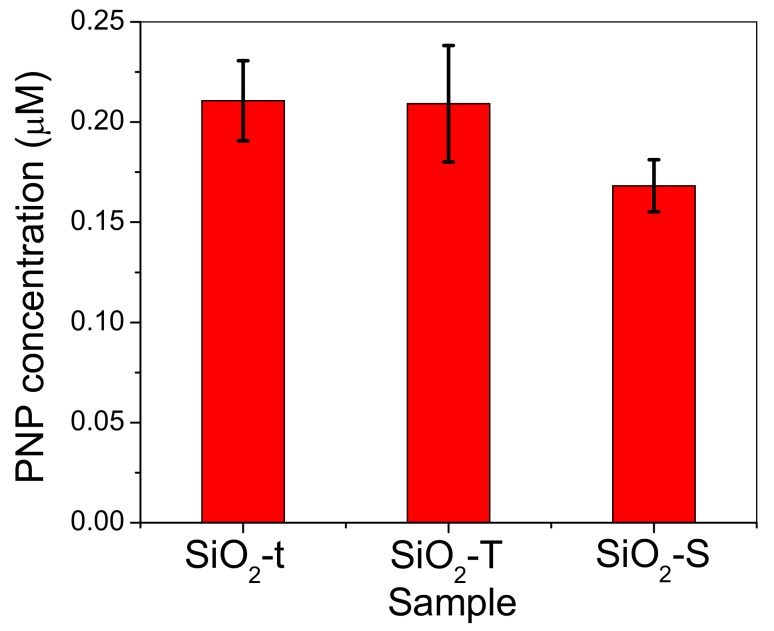
p-NP concentration (μM) resulted from p-NPA hydrolysis reaction conducted in the presence of immobilized lipase on SiO_2_ samples. The reaction was performed in triplicate, error bars representing the deviation for three catalytic independent runs.

The UV VIS data presented in Figure 13 show typical curves for the concentration of the p-nitrophenol reaction product (denoted as p-NP) generated by p-NPA hydrolysis after 1h of incubation in the presence of immobilized lipase on SiO_2_. Thus, Figure 14 illustrates the higher hydrolysis ability of hybrid complexes based on silica with a tubular morphology compared to spherical ones, in terms of p-NP μM concentration, which is in line with TOC results. On the other hand, the lipase loaded on SiO_2_-t highlights the higher catalytic activity despite its much lower degree of immobilization. We could explain this by an apparent higher ability of thinner tubes to activate the lipase due to its confinement inside the tubes, which was confirmed by post immobilization BET analysis (Figure 4d).

**Figure 15:**
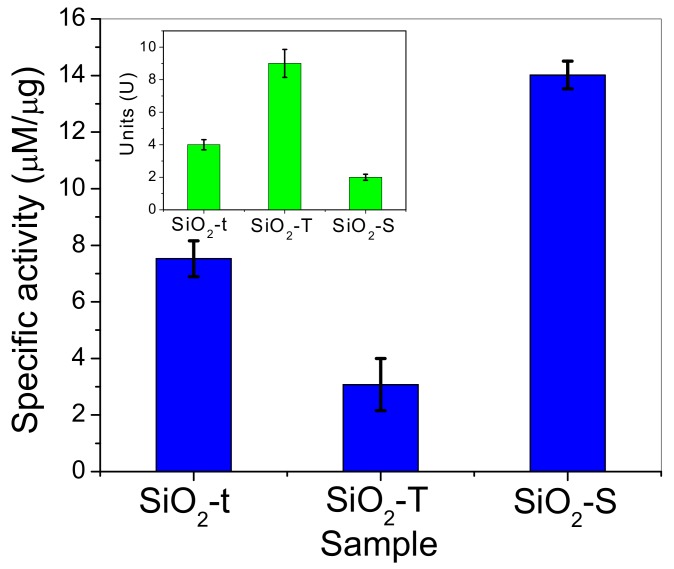
Specific activity (μM/μg) of immobilized lipase on SiO_2_ samples for p-NPA hydrolysis reaction. Inset: Biological activity of loaded lipase expressed in Units; 1 lipase unit (U) represents the amount (mg) of enzyme liberating 1 μmol p-NP per minute. All experiments were done in triplicate.

If catalytic activity is evaluated as a specific activity (see Figure 15, μM reaction product/μg of immobilized enzymes) we should note the following activity sequence: SiO_2_-S > SiO_2_-t > SiO_2_-T. A better specific activity of the lipase loaded on spherical particles is related to the inherent diffusional limitations near the enzymatic sites, especially inside the nanotubes, with the possibility of better contact with reactant of the lipase immobilized on spherical particles being an advantage. However, the small recorded lipase loading on SiO_2_-S reduces its catalytic performance. The inset shows the biological activity expressed in units (U), 1 lipase unit (U) represents the amount (mg) of enzyme liberating 1 μmol p-NP per minute, of the loaded lipase in agreement with TOC results (Figure 11).

These catalytic tests describe the enzymatic activity of a hybrid complex as dependent on morphology. A higher enzyme loading was obtained for larger tubes (SiO_2_-T), a greater amount of product was achieved by using the thinner tubes (SiO_2_-t) and the greatest specific enzymatic activity was recorded for spherical particles (SiO_2_-S).

Our results can be compared to the related literature, which indicates that the activation of adsorbed lipase onto solid surfaces is strongly dependent on the surrounding medium characteristics. Tran et al. [9] found that increased hydrophobicity is beneficial for lipase activity but not in aqueous mediums, and also it seems that the hydrophobic/hydrophilic nature of the solid carrier can induce the activation of lipase lid/flap [27] which should result in increased activity.

Moreover, regarding the enzymatic activity, the tubular morphology seems to be suitable for valuable hybrid complex development because the empty, long tubes ensure the appropriate conformation flexibility of the immobilized lipase.

Together, these results are relevant for considering the catalytic activity of immobilized lipase onto SiO_2_ and include the following parameters: the degree of immobilization (which, according to our results is strongly dependent on morphology and textural characteristics of inorganic matrix), the enzyme activation by the engineered microenvironment (hydrophilic/hydrophobic nature of the vicinal inorganic surface), the possible denaturation (loss of conformational flexibility or induced spatial restrictions by the solid host) and leaching in solution post immobilization, and the accessibility of the substrate to the active enzymatic sites. 

According to these considerations, since the enzymatic activity is significant for lipase immobilized on SiO_2_-t, the synthesis protocol of the SiO_2_-t matrix, the enzyme immobilization and enzymatic reaction parameters are worth optimizing in order to achieve and exploit a better enzyme–SiO_2_ hybrid structure (higher payload, improved enzymatic stability).

## 3. Materials and Methods

### 3.1. Materials

#### 3.1.1. Synthesis of SiO_2_ Matrices

The SiO_2_-T synthesis has been reported earlier [16]. In accordance with the synthesis of tubular SiO_2_ previously made by Nakamura [15], tetraorthosilicate (TEOS) was used as the silicon source (Alfa Aesar, Haverhill, MA, USA, 99%), with absolute ethanol (Riedel de Haen, Seelze, Germany) as a solvent, and ammonia (25% *w*/*w* aq. Alfa Aesar) and DL tartaric acid (TA) as the templating agent. A 50 mL mixture containing the above mentioned components was obtained according to the following molar ratio: 1(TEOS):0.07(Tartaric Acid):43(Ethanol):36(H_2_O), at 20 °C. The ammonia (25%) was added slowly (0.8 mL/min), under gentle stirring, in order to obtain a white suspension which was left to age for one hour.

By using similar conditions, and instant addition of 15 mL ammonia (25%) to the silica sol, the spherical morphology (SiO_2_-S) is obtained. 

In the case of SiO_2_-t, the previous molar ratio is slightly modified according to the following values, 1(TEOS):0.03(Tartaric Acid):18(Ethanol):10(H_2_O), which means a higher concentration of the starting solution, while the working temperature was decreased to 0 °C.

For all samples, the resulting white precipitate was washed, filtered and dried at 100 °C for 3 h and then calcined in air at 500 °C for 3 h.

#### 3.1.2. The Formation of the Lipase-SiO_2_ Hybrid Structure

A 5 mL solution of lipase (0.8 mg/mL) (*Rhizopus orizae*, Sigma Aldrich, Saint Louis, MI, USA, 10 U/mg) in potassium phosphate buffer at pH 6.3 (K_2_HPO_4_ × 3 H_2_O > 99% and H_3_PO_4_ Sigma Aldrich) was added to SiO_2_ powder (0.02 g) previously dispersed in phosphate buffer and the resulting mixture was gently stirred for 24 h, at 10 °C. The suspension was centrifuged and the total organic carbon from the resulting supernatant solution was analyzed with N/C 2100 S Analytic Yena equipment (Analytik, Jena, Germany), the presence of chemical bonds specific to lipase being evidenced by FTIR-ATR characterization.

The SiO_2_ powders separated by centrifugation were firstly washed with potassium phosphate buffer (pH 6.3) and then with ultrapure water and dried at vacuum for FTIR-ATR measurements, re-dispersed in pure water for PL characterization or in Dimethyl Sulfoxide (DMSO, Sigma Aldrich) for catalytic activity investigation.

#### 3.1.3. Enzymatic Assays

A quantity of 0.002 g from each SiO_2_ matrix (SiO_2_-T loaded with 0.063 mg lipase, SiO_2_-t loaded with 0.028 mg lipase and SiO_2_-S loaded with 0.011 mg lipase) were dispersed in 2 mL mixture containing 0.0033 g p-NPA previously dissolved in 0.5 mL dimethyl sulfoxide (DMSO) and 1.5 mL phosphate buffer at pH 7.8. After 1 h of incubation at 37 °C, the liquid samples (supernatant) were separated by centrifugation and measured by UV-VIS. The corresponding values resulted from our tests for EU present in 0.002 g of each SiO_2_ matrices are: 0.11 EU (SiO_2_-t), 0.57 EU (SiO_2_-T) and, respectively 0.02 EU (SiO_2_-S). All experiments were performed in triplicate.

### 3.2. Methods

Scanning electron microscopy (SEM) was performed with a high-resolution microscope, FEI Quanta 3D FEG model, using a 5 kV voltage and an Everhart–Thornley secondary electron (SE) detector.

The Fourier-transform infrared (FT-IR) spectra of the pristine SiO_2_ samples were obtained using a Nicolet Spectrometer Nico 6700 FT-IR between 400 and 4000 cm^−1^ and the KBr pellet technique.

In order to evaluate and compare the surface areas, pore volume and pore distribution of the samples, nitrogen sorption measurements were done using a Micromeritics ASAP 2020 (Norcross, GA, USA) automated gas sorption system, at −196 °C. Specific surface areas (S_BET_) were calculated with the Brunauer–Emmett–Teller (BET) based on the adsorption data recorded in the relative pressure domain of 0.05–0.30. By using the adsorption data the total pore volume (V_total_) was found. The pore size distribution curves were obtained from the desorption branch data correlated with the Barrett–Joyner–Halenda (BJH) method.

FTIR-ATR spectra were recorded with a Tensor 27 Bruker system (Nijmegen, Germany). The device is equipped with a DTGS detector equipped with KBr beam-splitter and a mid-IR source, the integral measurement range being 4000–400 cm^−1^. The resolution of the measurements was 1 cm^−1^. The sample cell is Pike Miracle’s single-bounce attenuated total reflectance (ATR), equipped with a unique ZnSe crystal. All measurements were performed at room temperature, under the same conditions, regardless of the sample form (liquid or solid). Device settings: 32-scanned accumulation with initial background scanning, atmospheric compensation recording (CO_2_/water vapor) 6 mm main slit, RT-DLaTGS detector set run, scanner speed set for 10 KHz.

Fluorescence measurements were carried out to assess the lipase immobilization by the nanoparticles. The emission of the main intrinsic fluorophore, that is tryptophan residue (Trp), is recorded with an excitation wavelength of 270 nm at a scan rate of 100 nm min^−1^ and a spectral resolution of 0.5 nm using a fluorescence spectrometer (Jasco FP 6500 and Carry Eclipse, ALT, East Lyme, CT, USA) with slits set at 5 nm in excitation and 5 nm in emission. All fluorescence measurements were performed at 25 °C.

TOC Analysis was performed with a N/C 2100S Analytic Yena equipment. For the analysis of organic carbon (non-purging organic carbon), the samples were acidified with 2 mol/L HCl and then bubbled for 2 min with 5.0 oxygen to remove any inorganic carbon traces. The autosampler syringe automatically injected a 500 μL volume into the furnace located in the analyzer containing a platinum catalyst and the NDIR detector.

Electrokinetic potential measurements were carried out on a Malvern Nano ZS Zetasizer, Model ZEN 3600 at room temperature. Each mean value of the electrophoretic mobility comprises 20 measurement cycles and the ξ values were calculated by means of the Helmholtz-Smoluchowski equation. Note that this approach is only rigorously valid for spherical particles and deviations within 20 % of the true values may occur for rod-like nanostructures [36]. As we are mainly interested in the scaling of the lipase immobilization with ξ values, the approximation used for treating the electrophoretic mobility could be considered beyond the purpose of the present investigation.

UV-VIS spectra for p-NP formation were registered with an Analytic Jena Specord 2000 spectrophotometer (Analytik, Jena, Germany).

## 4. Conclusions

The above presented results regarding the synthesis, characterization and enzymatic functionalization of tubular and spherical silica matrices are in good agreement with the latest attempts to design affordable and efficient hybrid enzyme-inorganic carriers. 

The lipase immobilization and its subsequent catalytic activity evaluation were performed involving tubular SiO_2_-T (diameters of 200–300 nm), spherical SiO_2_-S (both morphologies, SiO_2_-T and SiO_2_-S, being previously reported), as well as a newly synthesized nanotubes (SiO_2_-t) with an empty core and smaller diameters (20–50 nm).

The product of the p-nitrophenyl acetate hydrolysis reaction was studied in order to identify the enzymatic activity sequence of the developed lipase–SiO_2_ hybrid structures.

The highest amount of p-NP (hydrolysis reaction product) was obtained in the presence of immobilized lipase on thinner tubes (SiO_2_-t) despite the greater amount of lipase loaded on larger ones (SiO_2_-T).

## Figures and Tables

**Table 1:** Band assignation from FTIR obtained for the SiO_2_ samples prior to lipase immobilization.

Wavenumber (cm^−1^)	Assignation
1124	Si-O stretching in SiO_2_ [16]
~1200 (shoulder)	asymmetric vibration of Si-O-Si [19]
~980–960 (shoulder)	silanol groups (Si-OH) [21]
847	ν_s(Si-O-Si)_ [20]
484	δ_(Si-O-Si)_ [20]
1650	H_2_O [20,28]
3350–3600	structural hydroxyls and free OH groups [22,28]

**Table 2:** FTIR bands assignation for lipase.

Identified Band (cm^−1^)	Assignment
3271	N-H *(γ)*
2930	C-H *(γ)*
1635	Amide I, N-H def., (*δ*)
1521	Amide II, N-H stretch., (*γ*)
1245	Amide III, C-N stretch., (*γ*)
1060	C-O-C *(γ)*
1395	C-OH bending
650–600	C-C bending
